# Supplementary figures and images for: Reconstructed Genome-Scale Metabolic Model Characterizes Adaptive Metabolic Flux Changes in Peripheral Blood Mononuclear Cells in Severe COVID-19 Patients
Source: Int J Mol Sci. 2022 Oct 17;23(20):12400. doi: 10.3390/ijms232012400 (PMC9604493; doi:10.3390/ijms232012400)

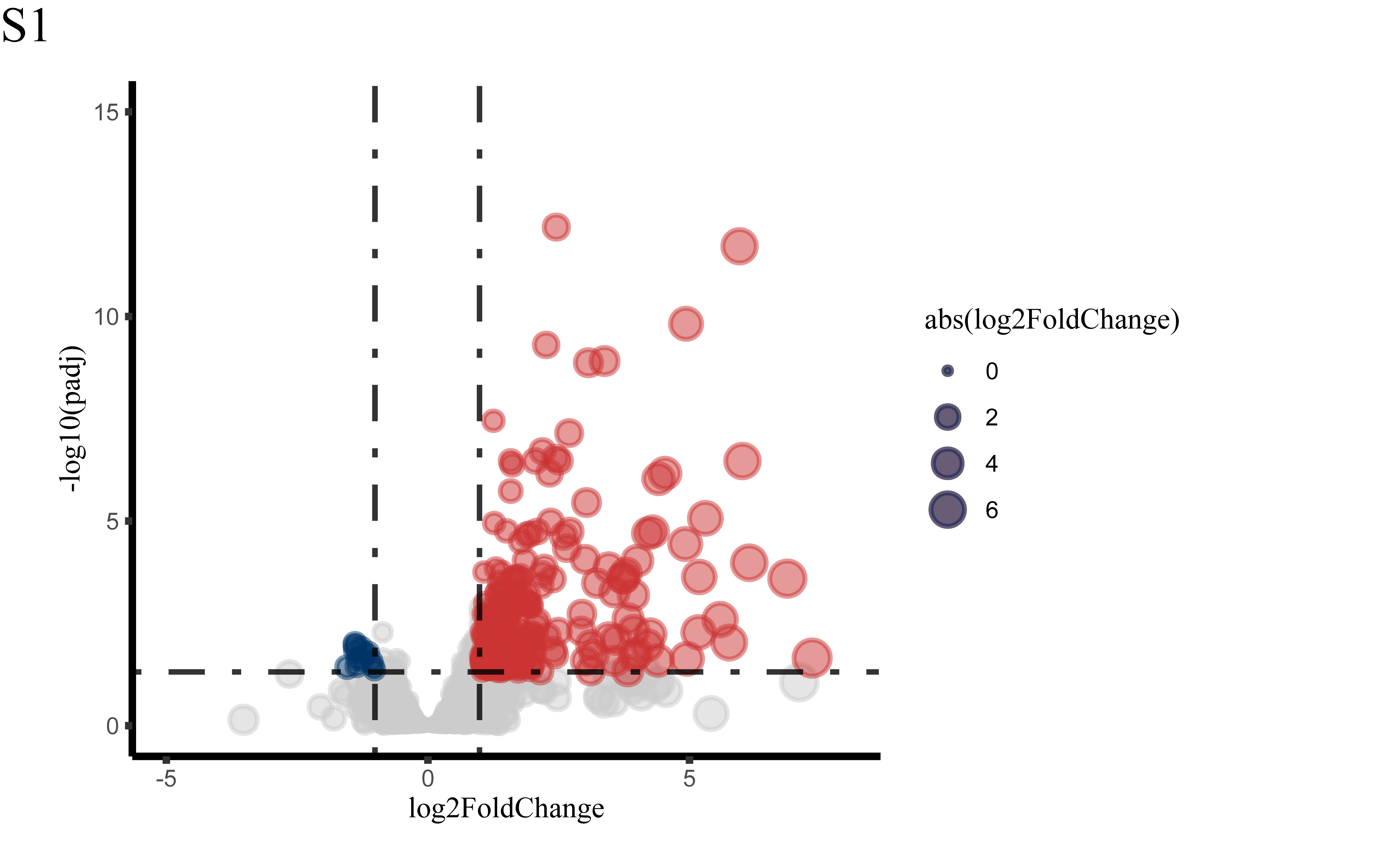

Supplement: Supplementary file 1 [file ijms-23-12400-s001.zip › Fig S1.tif]
